# Hypothesis: bipolar disorder is an Epstein–Barr virus‐driven chronic autoimmune disease – implications for immunotherapy

**DOI:** 10.1002/cti2.1116

**Published:** 2020-04-06

**Authors:** Michael P Pender

**Affiliations:** ^1^ Faculty of Medicine The University of Queensland Brisbane QLD Australia; ^2^ Department of Neurology Royal Brisbane and Women's Hospital Brisbane QLD Australia

**Keywords:** autoimmune, bipolar disorder, CD8 T cell, Epstein–Barr virus, sunlight, T‐cell therapy

## Abstract

Bipolar disorder (BD) is a chronic disease characterised by episodes of major depression and episodes of mania or hypomania, with a worldwide prevalence of 2.4%. The cause of BD is unknown. Here, I propose the hypothesis that BD is a chronic autoimmune disease caused by Epstein–Barr virus (EBV) infection of autoreactive B cells. It is postulated that EBV‐infected autoreactive B cells accumulate in the brain where they provide costimulatory survival signals to autoreactive T cells and differentiate into plasma cells producing pathogenic autoantibodies targeting brain components such as the N‐methyl‐D‐aspartate receptor. It is also proposed that the accumulation of EBV‐infected autoreactive B cells in the brain is a consequence of a genetically determined defect in the ability of CD8^+^ T cells to control EBV infection. The theory is supported by studies indicating that autoimmunity, EBV infection and CD8^+^ T‐cell deficiency all have roles in the pathogenesis of BD. According to the hypothesis, BD should be able to be treated by EBV‐specific T‐cell therapy and to be prevented by vaccination against EBV in early childhood. Exposure to sunlight or appropriate artificial light should also be beneficial in BD by augmenting CD8^+^ T‐cell control of EBV infection.

## Graphical abstract

This article proposes the novel hypothesis that bipolar disorder (BD) is a chronic autoimmune disease caused by Epstein–Barr virus (EBV) infection of autoreactive B cells, which accumulate in the brain where they facilitate an autoimmune attack on brain components such as the NMDA receptor. It is postulated that the accumulation of EBV‐infected autoreactive B cells in the brain is a consequence of a genetically determined defect in the ability of CD8^+^ T cells to control EBV infection. According to the hypothesis, BD should be able to be treated by EBV‐specific T‐cell therapy and to be prevented by vaccination against EBV early in life.

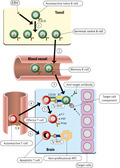

## Introduction

Bipolar disorder (BD) is a chronic disease characterised by episodes of major depression and episodes of mania or hypomania,^1^ with a worldwide prevalence of 2.4% when subthreshold BD is included.^2^ The cause of BD is unknown. The concordance rate of BD in monozygotic twins is 40–70%,^3^ indicating that genetic factors have a major role in the development of the disease. Given that this concordance rate is substantially less than 100%, environmental factors are also likely to make a significant contribution to the pathogenesis of the disease. There is some evidence that BD is a chronic autoimmune disease. I have previously hypothesised that all human chronic autoimmune diseases are caused by Epstein–Barr virus (EBV) infection of autoreactive B cells, which accumulate in the target organ where they provide costimulatory survival signals to autoreactive T cells and differentiate into plasma cells producing pathogenic autoantibodies.^4^ Here, I hypothesise that BD is an EBV‐driven chronic autoimmune disease. The hypothesis gives rise to predictions that can be tested. A crucial prediction derived from the hypothesis is that BD should be able to be treated by EBV‐specific T‐cell therapy and to be prevented by vaccination against EBV early in life. Exposure to sunlight or appropriate artificial light should also be beneficial in BD by augmenting CD8^+^ T‐cell control of EBV infection.

The article will proceed through the following sections: (1) the pathophysiology of BD; (2) evidence that BD is an autoimmune disease; (3) introduction to EBV; (4) introduction to EBV‐infected autoreactive B‐cell hypothesis of autoimmunity; (5) evidence for a role of EBV in the pathogenesis of BD; (6) evidence for a role of CD8^+^ T‐cell deficiency in BD; (7) the benefit of sunlight; (8) the role of stress; (9) proposed hypothesis for the development of BD; (10) testing the hypothesis, including the implications for immunotherapy and phototherapy; and (11) conclusion.

## Pathophysiology of BD

The pathophysiology of BD is complex and incompletely understood. Functional magnetic resonance imaging studies of emotional processing in BD have demonstrated underactivity of the ventrolateral prefrontal cortex and overactivity in the limbic regions, thalamus and basal ganglia.^5^ Diffusion tensor imaging studies of the brain in BD have shown decreased fractional anisotropy, indicating decreased structural connectivity, in the corpus callosum.^6^, ^7^ Histological studies of the brain in BD have revealed a decrease in hippocampal N‐methyl‐D‐aspartate (NMDA) receptors with open ion channels, but no decrease in kainate or AMPA receptors.^8^ Consistent with these findings, there is also a decreased expression of transcripts for the NR1 and NR2A subunits of the NMDA receptor and for SAP102, an NMDA receptor‐interacting protein, in the hippocampus in BD.^9^ It is possible that the decrease in hippocampal NMDA receptors in BD is caused by the anti‐NMDA receptor immunoglobulin G (IgG) antibodies that have been detected in individuals with mania.^10^

The beneficial effect of lithium in bipolar disorder has been attributed to its ability to inhibit glutamatergic neurotransmission mediated through NMDA receptors.^11^, ^12^ It has been proposed that in psychoses, such as bipolar disorder, hypofunctional NMDA receptors on inhibitory GABAergic hippocampal interneurons cause underactivity of the interneurons, which then release less GABA, which in turn disinhibits the pyramidal neurons in the hippocampus so that they release more glutamate from their projections to the midbrain and corpus striatum.^13^ This explanation could account for the apparent paradox that lithium, an agent thought to block glutamatergic neurotransmission,^11^ is beneficial in a disease, BD, in which there is a decrease in hippocampal NMDA receptors.^8^

## Evidence that BD is an autoimmune disease

One important clue to the likelihood of BD being an autoimmune disease comes from the observations that, like multiple sclerosis (MS), which is a chronic autoimmune disease of the central nervous system (CNS), BD tends to present clinically in adolescence and early adulthood and that it initially has a relapsing–remitting course, which later becomes progressive and unremitting.^14^ Intrathecal IgG synthesis, a hallmark of MS, occurs in 30% of patients with BD compared with 4% of controls,^15^ as well as in healthy siblings of MS patients.^16^ It has been hypothesised that intrathecal IgG synthesis with oligoclonal bands is caused by clonal expansion of EBV‐infected autoreactive B cells in the CNS.^4^ Increased serum/plasma interleukin‐6 levels in BD^7^, ^17^, ^18^ provide further evidence for a role of the immune system in the pathogenesis of BD.

Another indication that BD is an autoimmune disorder is the concurrence of BD with other autoimmune diseases. One study found that 28.3% of patients with BD had autoimmune thyroiditis, as determined by the presence of anti‐thyroperoxidase antibodies, compared with 13.5% of the general population.^19^ In particular, MS is strongly associated with BD and major depressive disorder – Carta and colleagues found that 46.7% of patients with MS had a mood disorder, 26.3% had major depressive disorder, and 9.9% had bipolar spectrum disorder, compared with 5.2%, 4.6% and 0.25% of controls, respectively.^20^ Furthermore, patients with coeliac disease have substantially higher prevalences of major depressive disorder and BD than do controls.^21^ Conversely, patients with BD have increased serum anti‐gliadin IgG antibodies.^22^ There is also an increased frequency of BD in each of the following autoimmune diseases: pemphigus,^23^ systemic lupus erythematosus,^24^, ^25^ rheumatoid arthritis,^25^ autoimmune vasculitis,^25^ Sjögren's syndrome^25^ and Crohn's disease.^25^ Moreover, the female children of parents with BD have an increased prevalence of autoimmune thyroiditis.^26^ The first‐degree relatives of individuals with schizophrenia, a possible autoimmune disease, have not only an increased risk of developing schizophrenia but also an increased risk of developing BD.^27^ It is important to note that the above studies show that there is an immune dysregulation in a subset of patients with BD; they do not show that all patients with BD have immune dysregulation.

A key question is, if BD is an autoimmune disease, what is the target autoantigen? One obvious potential autoantigen is the NMDA receptor, which is decreased in the hippocampus in BD.^8^ Support for the role of autoimmunity to the NMDA receptor in the pathogenesis of BD is provided by the observation that some patients presenting with mania have subsequently been diagnosed as having anti‐NMDA receptor encephalitis,^28^, ^29^, ^30^ which is an autoimmune disease of the CNS mediated by antibodies targeting the NR1 subunit of the NMDA receptor and leading to the internalisation of the receptor.^31^ Importantly, patients with acute mania have been found to have increased serum IgG antibodies to the NR2 subunit of the NMDA receptor; these antibodies had declined substantially at follow‐up 6 months later.^10^ It remains to be determined whether these antibodies directed against the NR2 subunit of the NMDA receptor are pathogenic. An autoimmune process in BD might also target brain components other than the NMDA receptor, such as molecules associated with the NMDA receptor and its signalling pathway.

## Introduction to EBV

**Figure 1:**
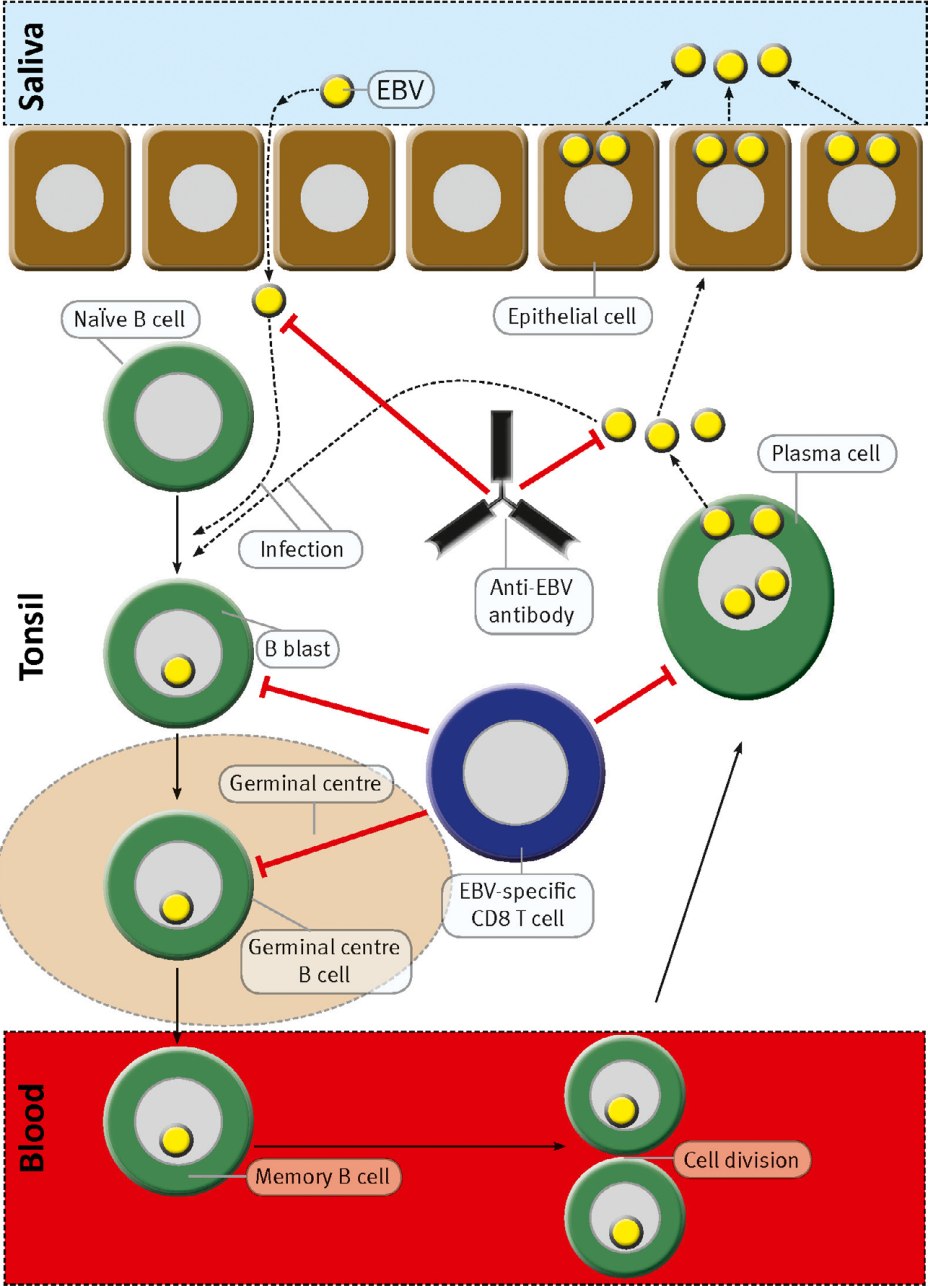
Normal sequence of events during infection of the tonsil by EBV. During primary infection, EBV enters the tonsil from the saliva and infects naïve B cells, driving them out of the resting state to become activated proliferating B blasts. The B blasts enter germinal centres where they proliferate intensely and differentiate into latently infected memory B cells, which then exit from the tonsil and circulate in the blood. The infected memory B cells do not express any viral proteins except during cell division when they express EBNA1. When latently infected memory B cells returning to the tonsil differentiate into plasma cells, the lytic phase of infection is initiated and free virus particles (virions) are produced. The virions infect tonsil epithelial cells where the virus replicates at a high rate and is shed into saliva for transmission to new hosts. Newly formed virions also infect additional naïve B cells in the same host, thereby completing the cycle necessary for the persistence of EBV in a lifelong infection. During primary infection, this cycle initially proceeds unchecked by the immune system. However, the infected host soon mounts an immune response against the virus. EBV‐specific cytotoxic CD8^+^ T cells kill infected cells expressing viral proteins, and anti‐EBV antibodies neutralise viral infectivity by binding to free virus. Red lines with perpendicular bars indicate inhibition. This model is based on the work published by Thorley‐Lawson and colleagues.^32^, ^33^, ^34^, ^91^ Modified from Pender^62^ through a Creative Commons Licence.

Epstein–Barr virus is a ubiquitous double‐stranded DNA γ‐herpesvirus infecting 90–95% of the adults in the world. EBV is the only human virus that infects, activates, clonally expands and persists latently in B lymphocytes for the duration of the infected individual's life. To achieve this, EBV exploits the normal B‐cell differentiation pathways.^32^ In primary infection, EBV enters the tonsil from saliva and infects naïve B cells, forcing them out of the resting state so that they become activated proliferating B blasts. The B blasts then enter a germinal centre where they pass through a germinal centre reaction and differentiate into latently infected memory B cells that circulate in the blood^32^ (Figure 1). On returning to the tonsil, latently infected memory B cells can differentiate into plasma cells, which initiates the lytic phase of infection.^33^ This culminates in the generation of virions. The newly formed virions infect epithelial cells in the tonsil where the virus reproduces rapidly and is continuously released into saliva to be transmitted to new hosts.^34^ The new virions also infect naïve B cells in the same host in order to complete the cycle required for EBV to persist as a lifelong infection.^35^ EBV deploys a series of different transcription programmes in order to pass through the discrete stages of its life cycle.^32^ In order to activate the blast phase after infecting naïve B cells, EBV uses the latency III transcription programme to express all its latent proteins, namely Epstein–Barr nuclear antigen 1 (EBNA1), EBNA2, EBNA3A, EBNA3B, EBNA3C and EBNALP, and latent membrane protein 1 (LMP1), LMP2A and LMP2B. The infected blast enters a germinal centre where it turns off expression of EBNA2, EBNA3A, EBNA3B, EBNA3C and EBNALP and continues to express EBNA1, LMP1 and LMP2 (latency II transcription programme). During its passage through the germinal centre phase, the EBV‐infected B blast differentiates into a memory B cell. Latently infected memory B cells cannot be detected by EBV‐specific T cells or antibodies because they express no viral proteins except during homeostatic cell division, when they express only EBNA1 (latency I transcription programme), which is required to duplicate the EBV genome and transmit it to daughter cells. In order to generate new infectious virions, EBV infection becomes reactivated through the lytic transcription programme when latently infected memory B cells differentiate into plasma cells.^33^

In healthy individuals, EBV infection is rigorously controlled by EBV‐specific immune responses. In particular, cytotoxic CD8^+^ T cells play a major role in regulating EBV infection. By targeting EBV‐encoded latent and lytic proteins, EBV‐specific CD8^+^ T cells kill proliferating and lytically infected cells, respectively^36^, ^37^ (Figure 1).

## Introduction to the EBV‐infected autoreactive B‐cell hypothesis of autoimmunity

Genes contributing to the pathogenesis of chronic autoimmune diseases consist of those causing a general susceptibility to autoimmunity and those predisposing to specific autoimmune diseases. One common manifestation of a general susceptibility to autoimmunity is an increased frequency of other autoimmune diseases in individuals with a particular autoimmune disease and in their blood relatives.^38^, ^39^, ^40^, ^41^ It is likely that the general susceptibility to autoimmunity has a Mendelian‐dominant inheritance pattern.^38^ I have proposed that the general predisposition to autoimmunity is due to a genetic CD8^+^ T‐cell deficiency causing defective CD8^+^ T‐cell control of EBV infection.^42^ The best‐characterised genes causing susceptibility to specific autoimmune diseases are HLA class II and less frequently HLA class I alleles.^43^ It is likely that the corresponding HLA molecules determine the autoantigens and therefore the target organs, which are recognised by autoreactive T cells activated by cross‐reacting foreign antigens or modified autoantigens.

The EBV‐infected autoreactive B‐cell hypothesis of autoimmunity, which was published in 2003, proposes that EBV infection of autoreactive B cells causes human chronic autoimmune diseases.^4^, ^42^ It postulates that a genetic defect in CD8^+^ T‐cell control of EBV infection allows the accumulation of EBV‐infected autoreactive B cells in the target organ. It also postulates that EBV‐infected autoreactive B cells in the target organ provide costimulatory survival signals to autoreactive T cells that would otherwise undergo activation‐induced apoptosis and die in the target organ. Furthermore, it is proposed that the now‐surviving autoreactive T cells provide T‐cell help to the EBV‐infected autoreactive B cells, which then differentiate into plasma cells producing pathogenic autoantibodies. The likelihood of EBV infecting autoreactive naïve B cells is considerable because at least 20% of human naïve B cells are autoreactive.^44^ Several predictions derived from the hypothesis have subsequently been substantiated, namely the accumulation of EBV‐infected B cells and plasma cells in the brain in MS^45^, ^46^, ^47^, ^48^, ^49^; EBV infection of autoreactive memory B cells in acute infectious mononucleosis^50^; EBV infection of autoreactive plasma cells in the joints in rheumatoid arthritis^51^ and salivary glands in Sjögren's syndrome^52^; decreased CD8^+^ T‐cell immunity to EBV in MS^53^, ^54^; improvement of MS with rituximab, which kills EBV‐infected B cells and uninfected B cells^55^; and a beneficial effect of EBV‐specific T‐cell therapy in MS.^56^, ^57^

## Evidence for a role of EBV in the pathogenesis of BD

At present, the evidence for a role of EBV infection in the pathogenesis of BD is limited. Gotlieb‐Stematsky and colleagues found that the geometric mean titre of serum antibodies against EBV viral capsid antigen in 27 patients with primary affective disorders (depression and mania) was significantly higher than in 25 neurological disease controls (54.43 versus 16.47, respectively, *P* < 0.001).^58^ Another study found a direct correlation between the severity of depression in depressed patients and the titres of serum antibodies against EBV early antigen, but not viral capsid antigen.^59^ Haeri and colleagues found serological evidence of EBV reactivation more frequently in 100 pregnant women with depression than in 100 healthy pregnant women without depression^60^; this was interpreted by the authors as possibly being due to increased stress from the depression, but an alternative explanation is that the depression was caused by poorly controlled EBV infection. The high anti‐EBV antibody titres in BD might reflect a high number of EBV‐infected cells. It is interesting to note that clozapine, an atypical antipsychotic drug used to treat BD, inhibits EBV reactivation.^61^

## Evidence for a role of CD8^+^ T‐cell deficiency in BD

A key component of the EBV‐infected autoreactive B‐cell hypothesis of autoimmunity is that the accumulation of EBV‐infected autoreactive B cells in the target organ is attributed to a genetic defect in the ability of EBV‐specific CD8^+^ T cells to control EBV infection.^4^, ^42^ The most likely explanation for the defect is the CD8^+^ T‐cell deficiency that is a characteristic feature of the human chronic autoimmune diseases, including MS.^42^, ^62^ This CD8^+^ T‐cell deficiency is also present in the healthy blood relatives of patients with chronic autoimmune diseases, indicating that the deficiency is genetically determined.^42^ In MS, it has been shown that the CD8^+^ T‐cell deficiency is due to a decrease in both the CD8^+^ effector memory (EM) T cells and the CD8^+^ EM re‐expressing CD45RA (EMRA) T cells.^63^

It is therefore of great interest that patients with BD have a decreased frequency of circulating CD8^+^ T cells^7^, ^64^ and that the decrease in CD8^+^ T cells is due to a decrease in both CD8^+^ EM T cells and CD8^+^ EMRA T cells and is the most marked during the manic phase of bipolar disorder.^7^ Moreover, the decrease in CD8^+^ EMRA T cells was found to be directly correlated with decreased fractional anisotropy, indicating decreased connectivity, in the corpus callosum and corona radiata.^7^ It is likely that the deficiency of CD8^+^ T cells in BD impairs immune control of EBV, as it does in MS.^54^, ^65^

## Benefit of sunlight

Seasonal affective disorder is characterised by recurrent episodes of major depression in autumn and winter with spontaneous remission in spring and summer.^66^ Most individuals with seasonal affective disorder also have BD.^66^ Bright artificial light, particularly in the early morning, is effective in reducing winter depressive symptoms of seasonal affective disorder.^67^ Bright light therapy is also efficacious in bipolar depression.^68^ Furthermore, exposure to morning sunlight reduces depression in seasonal affective disorder^69^ and shortens the duration of hospitalisation for bipolar depression.^70^ The mechanism for the beneficial effect of phototherapy on depression is unclear but has been variously attributed to effects on circadian rhythm, melatonin secretion or serotonin uptake.^71^

Another possible mechanism whereby phototherapy (sunlight or artificial light) might be beneficial in BD is a light‐induced increase in the number of CD8^+^ T cells that are available to regulate EBV infection. Treatment in a solarium or exposure to natural sunlight increases the proportion of circulating CD8^+^ T cells and decreases the CD4/CD8 T‐cell ratio.^72^, ^73^, ^74^ This effect of light is likely to be mediated at least partly by vitamin D for the following reasons: (1) within the immune system, the cells expressing the greatest concentration of the vitamin D receptor are activated CD8^+^ T cells^75^; (2) vitamin D augments the mitogen‐induced proliferation of CD8^+^ T cells and lowers the CD4/CD8 ratio *in vitro* ^76^; (3) administration of vitamin D increases the CD8^+^ T‐cell count^77^; and (4) in vitamin D deficiency, the proportion of CD8^+^ T cells in the blood decreases and the CD4/CD8 ratio increases.^78^ Interestingly, T cells also have an intrinsic sensitivity to blue light, which increases T‐cell motility through a H_2_O_2_ signalling pathway.^79^

## Role of stress

Stress in the form of negative life events is strongly associated with increases in the severity of subsequent depression and mania.^80^ It is important to note that stress suppresses the normal function of the immune system. Psychological stress compromises the ability of CD8^+^ T cells to prevent the reactivation of latent herpes simplex virus type I infection.^81^ Furthermore, stress decreases the ability of CD8^+^ T cells to kill EBV‐infected B cells,^82^ thereby inducing EBV reactivation.^82^, ^83^, ^84^ Thus, stress might increase the number of EBV‐infected autoreactive B cells and plasma cells and therefore exacerbate BD, leading to increased stress, further suppression of CD8^+^ T‐cell control of EBV infection, increased EBV‐infected autoreactive B cells and plasma cells and further worsening of BD – a vicious circle, indeed.

## Proposed hypothesis for the development of BD

**Figure 2:**
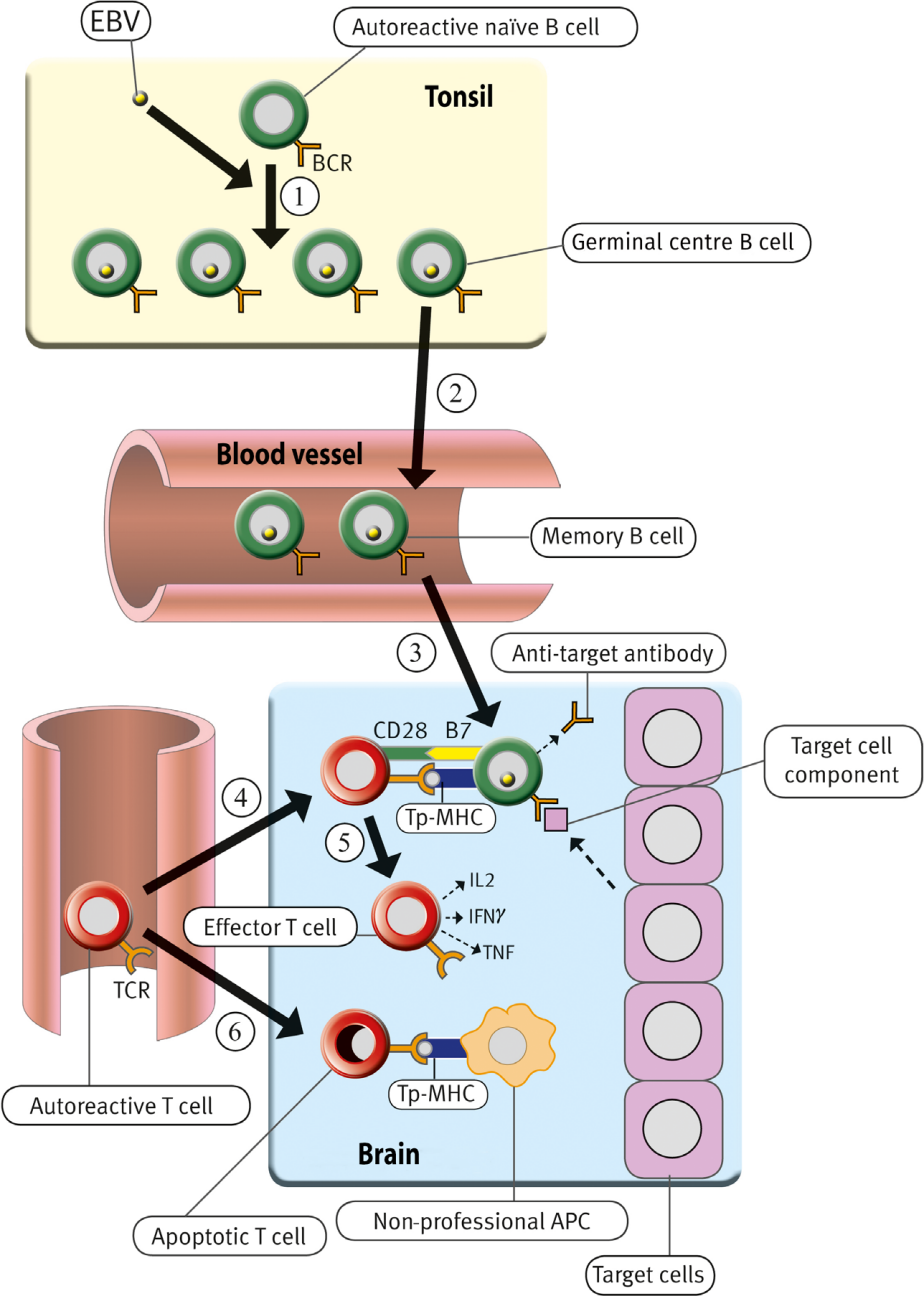
Proposed role of EBV infection in the development of BD. EBV infects autoreactive naïve B cells in the tonsil, driving them to proliferate and then enter germinal centres. In the germinal centres, they again proliferate and differentiate into latently infected autoreactive memory B cells (Step 1), which exit from the tonsil and circulate in the blood (Step 2). The number of EBV‐infected B cells is normally controlled by EBV‐specific cytotoxic CD8^+^ T cells, which kill the infected B cells. However, if there is a defective CD8^+^ T‐cell control of EBV infection, EBV‐infected cells survive and proliferate, resulting in an increased number of EBV‐infected autoreactive memory B cells that enter the brain where they lodge (Step 3). Circulating autoreactive T cells that have been activated in peripheral lymphoid organs enter the brain where they are reactivated by EBV‐infected autoreactive B cells presenting target cell peptides (Tp) bound to major histocompatibility complex (MHC) molecules (Step 4). These EBV‐infected B cells provide costimulatory survival signals (B7) to the CD28 receptor on the autoreactive T cells and therefore inhibit the activation‐induced T‐cell apoptosis, which normally occurs when autoreactive T cells enter the brain and interact with non‐professional antigen‐presenting cells (APC) that do not express B7 costimulatory molecules^92^, ^93^ (Step 6). After the autoreactive T cells have been reactivated by EBV‐infected autoreactive B cells, they produce cytokines such as interleukin‐2 (IL‐2), interferon‐γ (IFN‐γ) and tumour necrosis factor (TNF) and orchestrate an autoimmune attack on the target cells, such as neurons expressing the NMDA receptor (Step 5). The autoreactive T cells also provide T‐cell help to the EBV‐infected autoreactive B cells that then differentiate into plasma cells. These plasma cells produce pathogenic autoantibodies, which attack target cell components, such as NMDA receptors. BCR, B‐cell receptor; TCR, T‐cell receptor. Modified from Pender^42^ through a Creative Commons Licence.

It is proposed that the genes predisposing to BD can be subdivided into genes causing a general susceptibility to autoimmunity and genes causing specific susceptibility to BD. It is also postulated that the general susceptibility to autoimmunity is due to a genetic defect in the generation of the CD8^+^ T cells needed to regulate EBV infection.^42^ Based on the EBV‐infected autoreactive B‐cell hypothesis of autoimmunity,^4^, ^42^ it is proposed that the following sequence of events leads to the development of BD (Figure 2). EBV infection in people with a genetic defect in the generation of CD8^+^ T cells and a general predisposition to autoimmunity results in a high number of EBV‐infected autoreactive B cells, which infiltrate lymphoid and non‐lymphoid organs, including the CNS. In those people who also carry genes conferring specific susceptibility to BD, autoreactive T cells specific for CNS components, such as the NMDA receptor, are activated by cross‐reacting foreign antigens or modified autoantigens. These brain‐reactive T cells then enter the CNS where they receive costimulatory survival signals from the EBV‐infected autoreactive B cells lodged in the CNS. In turn, the now‐surviving autoreactive T cells provide T‐cell help to the EBV‐infected autoreactive B cells, which then differentiate into plasma cells producing pathogenic autoantibodies, including anti‐NMDA receptor antibodies. The autoantibodies interfere with glutamatergic or other neurotransmission in the CNS, thereby giving rise to the symptoms of BD.

## Testing the hypothesis

### Is EBV infection a prerequisite for the development of BD?

Patients with MS and patients with systemic lupus erythematosus are almost universally (99–100%) seropositive for EBV.^85^, ^86^ To determine whether EBV infection is a prerequisite for the development of BD, serological studies should be conducted in individuals with BD and, for comparison, healthy subjects. It is important that IgG reactivity to both EBNA1 and EBV viral capsid antigen is tested because some EBV‐infected individuals may be seronegative for one of these EBV antigens.

### Do EBV‐infected autoreactive B cells and plasma cells accumulate in the brain in BD?

Firstly, it should be determined whether there is an increased frequency of EBV‐infected B cells and plasma cells in the brain in BD compared with non‐autoimmune CNS disorders. Secondly, whether EBV‐infected B cells and plasma cells in the brain in BD are autoreactive could be addressed by determining whether they bind biotinylated NMDA receptor or other autoantigens in the same way that EBV‐infected plasma cells in the synovium bind citrullinated fibrinogen in rheumatoid arthritis.^51^

### Is the CD8^+^ T‐cell response to EBV decreased in BD?

Whether the CD8^+^ T‐cell response to EBV is decreased in BD could be addressed by immunological studies of the response of peripheral blood CD8^+^ T cells to EBV antigens in individuals with BD and in EBV‐seropositive healthy individuals, as have been used to demonstrate defective CD8^+^ T‐cell control of EBV infection in MS.^54^ It is also important to determine whether the CD8^+^ T‐cell deficiency in individuals with BD is also present in their healthy first‐degree relatives.

### Does the benefit of phototherapy in BD correlate with improved CD8^+^ T‐cell control of EBV?

To determine whether the clinical benefit of phototherapy in BD is related to improved CD8^+^ T‐cell control of EBV infection, the clinical effect of exposure to sunlight or artificial bright light could be correlated with any changes in the numbers of total CD8^+^ T cells, CD8^+^ EM T cells and CD8^+^ EMRA T cells and with any changes in the CD8^+^ T‐cell response to EBV after phototherapy. In the case of artificial light, it will be important to ensure that the light includes the UVB wavelengths (280–320 nm) needed to stimulate vitamin D synthesis in the skin.

### Does EBV vaccination protect against BD?

Epstein–Barr virus‐induced infectious mononucleosis, but not asymptomatic EBV infection, in healthy EBV‐seronegative young adults is effectively prevented by vaccination with recombinant EBV gp350 glycoprotein.^87^ The subjects who were vaccinated developed anti‐gp350 antibodies, which most likely provided the protective effect because monoclonal anti‐gp350 antibody neutralises EBV infectivity of B cells.^88^ The efficacy of this vaccine is limited because it protects only B cells, and not epithelial cells, from infection. Recently, it has been shown that vaccination of mice and non‐human primates with components of the glycoprotein gH/gL complex of the EBV viral‐fusion machinery potently neutralises infection of both epithelial cells and B cells.^89^ If BD truly is an EBV‐dependent chronic autoimmune disease, effective vaccination against EBV in early childhood should prevent BD.

### Does BD respond to therapy aimed at controlling EBV infection?

If BD is an EBV‐induced chronic autoimmune disease, there are two ways to control EBV infection specifically and therefore treat BD: (1) treatment with antiviral drugs; and (2) improving immunity to EBV. Aciclovir and related drugs have a limited therapeutic effect in EBV‐induced diseases because they inhibit EBV only in the lytic phase when it utilises its own DNA polymerase in order to replicate its DNA. They are ineffective in the latent phase when EBV employs EBNA1 to hijack host cell DNA polymerase to replicate its DNA. In the future, drugs targeting specific EBV proteins, such as small‐molecule inhibitors of EBNA1 DNA binding,^90^ have the potential to be more effective in controlling EBV infection.

In order to improve CD8^+^ T‐cell immunity to EBV, individuals with BD could be treated by intravenously infusing EBV‐specific cytotoxic CD8^+^ T cells after expansion *in vitro*, as in the treatment of patients with MS.^56^, ^57^ A potential risk of EBV‐specific T‐cell therapy is that, if EBV‐infected cells do accumulate in the brain in BD, the transferred T cells might increase inflammation in the brain and cause clinical deterioration. If BD is induced by EBV infection, EBV‐specific T‐cell therapy has the potential to be curative. Another way of improving immunity to EBV is EBV vaccination. An effective EBV vaccine, when it becomes available, might be beneficial even in patients with established BD. Vaccination against EBV also has the potential to increase inflammation in the brain and cause clinical deterioration.

## Conclusion

In conclusion, I have here proposed the novel hypothesis that BD is a chronic autoimmune disease caused by EBV infection of autoreactive B cells, which accumulate in the brain where they facilitate an autoimmune attack on brain components such as the NMDA receptor. It is postulated that the accumulation of EBV‐infected autoreactive B cells in the brain is a consequence of a genetically determined defect in the ability of CD8^+^ T cells to control EBV infection. According to the hypothesis, BD should be able to be treated by EBV‐specific T‐cell therapy and to be prevented by vaccination against EBV early in life. Exposure to sunlight or appropriate artificial light should also be beneficial in BD by augmenting CD8^+^ T‐cell control of EBV infection.

## Conflict of interest

The author declares no conflict of interest.

## References

[1] American Psychiatric Association . Diagnostic and Statistical Manual of Mental Disorders, 5th edn Arlington, VA: American Psychiatric Association; 2013.

[2] Merikangas KR , Jin R , He J‐P *et al* Prevalence and correlates of bipolar spectrum disorder in the World Mental Health Survey Initiative. Arch Gen Psychiatry 2011; 68: 241–251. 21383262 10.1001/archgenpsychiatry.2011.12 PMC3486639

[3] Craddock N , Jones I . Genetics of bipolar disorder. J Med Genet 1999; 36: 585–594. 10465107 10.1136/jmg.36.8.585 PMC1762980

[4] Pender MP . Infection of autoreactive B lymphocytes with EBV, causing chronic autoimmune diseases. Trends Immunol 2003; 24: 584–588. 14596882 10.1016/j.it.2003.09.005

[5] Delvecchio G , Fossati P , Boyer P *et al* Common and distinct neural correlates of emotional processing in bipolar disorder and major depressive disorder: a voxel‐based meta‐analysis of functional magnetic resonance imaging studies. Eur Neuropsychopharmacol 2012; 22: 100–113. 21820878 10.1016/j.euroneuro.2011.07.003

[6] Wise T , Radua J , Nortje G , Cleare AJ , Young AH , Arnone D . Voxel‐based meta‐analytical evidence of structural disconnectivity in major depression and bipolar disorder. Biol Psychiatry 2016; 79: 293–302. 25891219 10.1016/j.biopsych.2015.03.004

[7] Magioncalda P , Martino M , Tardito S *et al* White matter microstructure alterations correlate with terminally differentiated CD8^+^ effector T cell depletion in the peripheral blood in mania: Combined DTI and immunological investigation in the different phases of bipolar disorder. Brain Behav Immun 2018; 73: 192–204. 29723656 10.1016/j.bbi.2018.04.017

[8] Scarr E , Pavey G , Sundram S , MacKinnon A , Dean B . Decreased hippocampal NMDA, but not kainate or AMPA receptors in bipolar disorder. Bipolar Disord 2003; 5: 257–264. 12895203 10.1034/j.1399-5618.2003.00024.x

[9] McCullumsmith RE , Kristiansen LV , Beneyto M , Scarr E , Dean B , Meador‐Woodruff JH . Decreased NR1, NR2A, and SAP102 transcript expression in the hippocampus in bipolar disorder. Brain Res 2007; 1127: 108–118. 17113057 10.1016/j.brainres.2006.09.011 PMC2900828

[10] Dickerson F , Stallings C , Vaughan C , Origoni A , Khushalani S , Yolken R . Antibodies to the glutamate receptor in mania. Bipolar Disord 2012; 14: 547–553. 22672262 10.1111/j.1399-5618.2012.01028.x

[11] Hashimoto R , Hough C , Nakazawa T , Yamamoto T , Chuang D‐M . Lithium protection against glutamate excitotoxicity in rat cerebral cortical neurons: involvement of NMDA receptor inhibition possibly by decreasing NR2B tyrosine phosphorylation. J Neurochem 2002; 80: 589–597. 11841566 10.1046/j.0022-3042.2001.00728.x

[12] Basselin M , Chang L , Bell JM , Rapoport SI . Chronic lithium chloride administration attenuates brain NMDA receptor‐initiated signaling via arachidonic acid in unanesthetized rats. Neuropsychopharmacology 2006; 31: 1659–1674. 16292331 10.1038/sj.npp.1300920

[13] Lieberman JA , First MB . Psychotic disorders. N Engl J Med 2018; 379: 270–280. 30021088 10.1056/NEJMra1801490

[14] Berk M . Neuroprogression: pathways to progressive brain changes in bipolar disorder. Int J Neuropsychopharmacol 2009; 12: 441–445. 18922203 10.1017/S1461145708009498

[15] Stich O , Andres TA , Gross CM , Gerber SI , Rauer S , Langosch JM . An observational study of inflammation in the central nervous system in patients with bipolar disorder. Bipolar Disord 2015; 17: 291–302. 25109751 10.1111/bdi.12244

[16] Haghighi S , Andersen O , Rosengren L , Bergström T , Wahlström J , Nilsson S . Incidence of CSF abnormalities in siblings of multiple sclerosis patients and unrelated controls. J Neurol 2000; 247: 616–622. 11041329 10.1007/s004150070130

[17] Lu Y‐R , Rao Y‐B , Mou Y‐J *et al* High concentrations of serum interleukin‐6 and interleukin‐8 in patients with bipolar disorder. Medicine (Baltimore) 2019; 98: e14419. 30762747 10.1097/MD.0000000000014419 PMC6407988

[18] Wiener CD , Moreira FP , Portela LV *et al* Interleukin‐6 and interleukin‐10 in mood disorders: a population‐based study. Psychiatry Res 2019; 273: 685–689. 31207853 10.1016/j.psychres.2019.01.100

[19] Kupka RW , Nolen WA , Post RM *et al* High rate of autoimmune thyroiditis in bipolar disorder: lack of association with lithium exposure. Biol Psychiatry 2002; 51: 305–311. 11958781 10.1016/s0006-3223(01)01217-3

[20] Carta MG , Moro MF , Lorefice L *et al* The risk of bipolar disorders in multiple sclerosis. J Affect Disord 2014; 155: 255–260. 24295600 10.1016/j.jad.2013.11.008

[21] Carta MG , Conti A , Lecca F *et al* The burden of depressive and bipolar disorders in celiac disease. Clin Pract Epidemiol Ment Health 2015; 11: 180–185. 26962323 10.2174/1745017901511010180 PMC4763959

[22] Dickerson F , Stallings C , Origoni A *et al* Markers of gluten sensitivity and celiac disease in bipolar disorder. Bipolar Disord 2011; 13: 52–58. 21320252 10.1111/j.1399-5618.2011.00894.x

[23] Kridin K , Zelber‐Sagi S , Comaneshter D , Cohen AD . Bipolar disorder associated with another autoimmune disease—pemphigus: a population‐based study. Can J Psychiatry 2018; 63: 474–480. 29108425 10.1177/0706743717740344 PMC6099770

[24] Tiosano S , Nir Z , Gendelman O *et al* The association between systemic lupus erythematosus and bipolar disorder – a big data analysis. Eur Psychiatry 2017; 43: 116–119. 28525775 10.1016/j.eurpsy.2017.03.006

[25] Wang L‐Y , Chiang J‐H , Chen S‐F , Shen Y‐C . Systemic autoimmune diseases are associated with an increased risk of bipolar disorder: a nationwide population‐based cohort study. J Affect Disord 2018; 227: 31–37. 29049933 10.1016/j.jad.2017.10.027

[26] Hillegers MHJ , Reichart CG , Wals M *et al* Signs of a higher prevalence of autoimmune thyroiditis in female offspring of bipolar parents. Eur Neuropsychopharmacol 2007; 17: 394–399. 17140771 10.1016/j.euroneuro.2006.10.005

[27] Cheng C‐M , Chang W‐H , Chen M‐H *et al* Co‐aggregation of major psychiatric disorders in individuals with first‐degree relatives with schizophrenia: a nationwide population‐based study. Mol Psychiatry 2018; 23: 1756–1763. 29112198 10.1038/mp.2017.217

[28] Parratt KL , Allan M , Lewis SJG , Dalmau J , Halmagyi GM , Spies JM . Acute psychiatric illness in a young woman: an unusual form of encephalitis. Med J Aust 2009; 191: 284–286. 19740054 10.5694/j.1326-5377.2009.tb02787.x

[29] Kuo YL , Tsai HF , Lai MC , Lin CH , Yang YK . Anti‐NMDA receptor encephalitis with the initial presentation of psychotic mania. J Clin Neurosci 2012; 19: 896–898. 22330692 10.1016/j.jocn.2011.10.006

[30] Choe C‐u , Karamatskos E , Schattling B *et al* A clinical and neurobiological case of IgM NMDA receptor antibody associated encephalitis mimicking bipolar disorder. Psychiatry Res 2013; 208: 194–196. 23246244 10.1016/j.psychres.2012.09.035

[31] Dalmau J , Armangué T , Planagumà J *et al* An update on anti‐NMDA receptor encephalitis for neurologists and psychiatrists: mechanisms and models. Lancet Neurol 2019; 18: 1045–1057. 31326280 10.1016/S1474-4422(19)30244-3

[32] Thorley‐Lawson DA , Gross A . Persistence of the Epstein–Barr virus and the origins of associated lymphomas. N Engl J Med 2004; 350: 1328–1337. 15044644 10.1056/NEJMra032015

[33] Laichalk LL , Thorley‐Lawson DA . Terminal differentiation into plasma cells initiates the replicative cycle of Epstein–Barr virus *in vivo* . J Virol 2005; 79: 1296–1307. 15613356 10.1128/JVI.79.2.1296-1307.2005 PMC538585

[34] Hadinoto V , Shapiro M , Sun CC , Thorley‐Lawson DA . The dynamics of EBV shedding implicate a central role for epithelial cells in amplifying viral output. PLoS Pathog 2009; 5: e1000496. 19578433 10.1371/journal.ppat.1000496 PMC2698984

[35] Hawkins JB , Delgado‐Eckert E , Thorley‐Lawson DA , Shapiro M . The cycle of EBV infection explains persistence, the sizes of the infected cell populations and which come under CTL regulation. PLoS Pathog 2013; 9: e1003685. 24146621 10.1371/journal.ppat.1003685 PMC3798424

[36] Khanna R , Burrows SR . Role of cytotoxic T lymphocytes in Epstein–Barr virus‐associated diseases. Annu Rev Microbiol 2000; 54: 19–48. 11018123 10.1146/annurev.micro.54.1.19

[37] Hislop AD , Taylor GS , Sauce D , Rickinson AB . Cellular responses to viral infection in humans: lessons from Epstein–Barr virus. Annu Rev Immunol 2007; 25: 587–617. 17378764 10.1146/annurev.immunol.25.022106.141553

[38] Bias WB , Reveille JD , Beaty TH , Meyers DA , Arnett FC . Evidence that autoimmunity in man is a Mendelian dominant trait. Am J Hum Genet 1986; 39: 584–602. 3098096 PMC1684061

[39] McCombe PA , Chalk JB , Pender MP . Familial occurrence of multiple sclerosis with thyroid disease and systemic lupus erythematosus. J Neurol Sci 1990; 97: 163–171. 2401894 10.1016/0022-510x(90)90215-9

[40] Lin J‐P , Cash JM , Doyle SZ *et al* Familial clustering of rheumatoid arthritis with other autoimmune diseases. Hum Genet 1998; 103: 475–482. 9856493 10.1007/s004390050853

[41] Henderson RD , Bain CJ , Pender MP . The occurrence of autoimmune diseases in patients with multiple sclerosis and their families. J Clin Neurosci 2000; 7: 434–437. 10942666 10.1054/jocn.2000.0693

[42] Pender MP . CD8^+^ T‐cell deficiency, Epstein–Barr virus infection, vitamin D deficiency and steps to autoimmunity: a unifying hypothesis. Autoimmune Dis 2012; 2012: 189096. 22312480 10.1155/2012/189096 PMC3270541

[43] Thorsby E , Lie BA . HLA associated genetic predisposition to autoimmune diseases: genes involved and possible mechanisms. Transpl Immunol 2005; 14: 175–182. 15982560 10.1016/j.trim.2005.03.021

[44] Wardemann H , Yurasov S , Schaefer A , Young JW , Meffre E , Nussenzweig MC . Predominant autoantibody production by early human B cell precursors. Science 2003; 301: 1374–1377. 12920303 10.1126/science.1086907

[45] Serafini B , Rosicarelli B , Franciotta D *et al* Dysregulated Epstein–Barr virus infection in the multiple sclerosis brain. J Exp Med 2007; 204: 2899–2912. 17984305 10.1084/jem.20071030 PMC2118531

[46] Tzartos JS , Khan G , Vossenkamper A *et al* Association of innate immune activation with latent Epstein–Barr virus in active MS lesions. Neurology 2012; 78: 15–23. 22156987 10.1212/WNL.0b013e31823ed057

[47] Serafini B , Muzio L , Rosicarelli B , Aloisi F . Radioactive *in situ* hybridization for Epstein–Barr virus‐encoded small RNA supports presence of Epstein–Barr virus in the multiple sclerosis brain. Brain 2013; 136: e233. 23355688 10.1093/brain/aws315

[48] Hassani A , Corboy JR , Al‐Salam S , Khan G . Epstein–Barr virus is present in the brain of most cases of multiple sclerosis and may engage more than just B cells. PLoS One 2018; 13: e0192109. 29394264 10.1371/journal.pone.0192109 PMC5796799

[49] Moreno MA , Or‐Geva N , Aftab BT *et al* Molecular signature of Epstein–Barr virus infection in MS brain lesions. Neurol Neuroimmunol Neuroinflamm 2018; 5: e466. 29892607 10.1212/NXI.0000000000000466 PMC5994704

[50] Tracy SI , Kakalacheva K , Lünemann JD , Luzuriaga K , Middeldorp J , Thorley‐Lawson DA . Persistence of Epstein–Barr virus in self‐reactive memory B cells. J Virol 2012; 86: 12330–12340. 22951828 10.1128/JVI.01699-12 PMC3486485

[51] Croia C , Serafini B , Bombardieri M *et al* Epstein–Barr virus persistence and infection of autoreactive plasma cells in synovial lymphoid structures in rheumatoid arthritis. Ann Rheum Dis 2013; 72: 1559–1568. 23268369 10.1136/annrheumdis-2012-202352

[52] Croia C , Astorri E , Murray‐Brown W *et al* Implication of Epstein–Barr virus infection in disease‐specific autoreactive B cell activation in ectopic lymphoid structures of Sjögren's syndrome. Arthritis Rheumatol 2014; 66: 2545–2557. 24891330 10.1002/art.38726

[53] Pender MP , Csurhes PA , Lenarczyk A , Pfluger CMM , Burrows SR . Decreased T cell reactivity to Epstein–Barr virus infected lymphoblastoid cell lines in multiple sclerosis. J Neurol Neurosurg Psychiatry 2009; 80: 498–505. 19015225 10.1136/jnnp.2008.161018 PMC2663364

[54] Pender MP , Csurhes PA , Burrows JM , Burrows SR . Defective T‐cell control of Epstein–Barr virus infection in multiple sclerosis. Clin Transl Immunol 2017; 6: e126. 10.1038/cti.2016.87 PMC5292561 28197337

[55] Hauser SL , Waubant E , Arnold DL *et al* B‐cell depletion with rituximab in relapsing‐remitting multiple sclerosis. N Engl J Med 2008; 358: 676–688. 18272891 10.1056/NEJMoa0706383

[56] Pender MP , Csurhes PA , Smith C *et al* Epstein–Barr virus‐specific adoptive immunotherapy for progressive multiple sclerosis. Mult Scler 2014; 20: 1541–1544. 24493474 10.1177/1352458514521888 PMC4230458

[57] Pender MP , Csurhes PA , Smith C *et al* Epstein–Barr virus–specific T cell therapy for progressive multiple sclerosis. JCI Insight 2018; 3: e124714. 10.1172/jci.insight.124714 PMC6302936 30429369

[58] Gotlieb‐Stematsky T , Zonis J , Arlazoroff A , Mozes T , Sigal M , Szekely AG . Antibodies to Epstein–Barr virus, herpes simplex type 1, cytomegalovirus and measles virus in psychiatric patients. Arch Virol 1981; 67: 333–339. 6263228 10.1007/BF01314836

[59] Cooke RG , Warsh JJ , Hasey GM , McLaughlin BJM , Jorna T . Epstein–Barr virus antibodies and severity of depression. Biol Psychiatry 1991; 29: 621–623. 1647225 10.1016/0006-3223(91)90102-r

[60] Haeri S , Johnson N , Baker AM *et al* Maternal depression and Epstein–Barr virus reactivation in early pregnancy. Obstet Gynecol 2011; 117: 862–866. 21422857 10.1097/AOG.0b013e31820f3a30

[61] Anderson AG , Gaffy CB , Weseli JR , Gorres KL . Inhibition of Epstein–Barr virus lytic reactivation by the atypical antipsychotic drug clozapine. Viruses 2019; 11: E450. 10.3390/v11050450 PMC6563273 31108875

[62] Pender MP . The essential role of Epstein–Barr virus in the pathogenesis of multiple sclerosis. Neuroscientist 2011; 17: 351–367. 21075971 10.1177/1073858410381531 PMC3764840

[63] Pender MP , Csurhes PA , Pfluger CMM , Burrows SR . Deficiency of CD8^+^ effector memory T cells is an early and persistent feature of multiple sclerosis. Mult Scler 2014; 20: 1825–1832. 24842963 10.1177/1352458514536252 PMC4361480

[64] Barbosa IG , Rocha NP , Assis F *et al* Monocyte and lymphocyte activation in bipolar disorder: a new piece in the puzzle of immune dysfunction in mood disorders. Int J Neuropsychopharmacol 2014; 18: pyu021. 25539506 10.1093/ijnp/pyu021 PMC4368866

[65] Pender MP , Csurhes PA , Pfluger CMM , Burrows SR . CD8 T cell deficiency impairs control of Epstein–Barr virus and worsens with age in multiple sclerosis. J Neurol Neurosurg Psychiatry 2012; 83: 353–354. 21791511 10.1136/jnnp-2011-300213 PMC3277686

[66] Rosenthal NE , Sack DA , Gillin JC *et al* Seasonal affective disorder. A description of the syndrome and preliminary findings with light therapy. Arch Gen Psychiatry 1984; 41: 72–80. 6581756 10.1001/archpsyc.1984.01790120076010

[67] Terman M , Terman JS , Quitkin FM , McGrath PJ , Stewart JW , Rafferty B . Light therapy for seasonal affective disorder. A review of efficacy. Neuropsychopharmacology 1989; 2: 1–22. 2679625 10.1016/0893-133x(89)90002-x

[68] Yorguner Kupeli N , Bulut NS , Carkaxhiu Bulut G , Kurt E , Kora K . Efficacy of bright light therapy in bipolar depression. Psychiatry Res 2018; 260: 432–438. 29268206 10.1016/j.psychres.2017.12.020

[69] Wirz‐Justice A , Graw P , Kräuchi K *et al* ‘Natural’ light treatment of seasonal affective disorder. J Affect Disord 1996; 37: 109–120. 8731073 10.1016/0165-0327(95)00081-x

[70] Benedetti F , Colombo C , Barbini B , Campori E , Smeraldi E . Morning sunlight reduces length of hospitalization in bipolar depression. J Affect Disord 2001; 62: 221–223. 11223110 10.1016/s0165-0327(00)00149-x

[71] Parry BL , Maurer EL . Light treatment of mood disorders. Dialogues Clin Neurosci 2003; 5: 353–365. 22033495 10.31887/DCNS.2003.5.4/bparry PMC3181775

[72] Hersey P , Bradley M , Hasic E , Haran G , Edwards A , McCarthy WH . Immunological effects of solarium exposure. Lancet 1983; 321: 545–548. 10.1016/s0140-6736(83)92808-8 6131254

[73] Hersey P , Haran G , Hasic E , Edwards A . Alteration of T cell subsets and induction of suppressor T cell activity in normal subjects after exposure to sunlight. J Immunol 1983; 31: 171–174. 6223071

[74] Falkenbach A , Sedlmeyer A . Travel to sunny countries is associated with changes in immunological parameters. Photodermatol Photoimmunol Photomed 1997; 13: 139–142. 9453082 10.1111/j.1600-0781.1997.tb00217.x

[75] Veldman CM , Cantorna MT , DeLuca HF . Expression of 1,25‐dihydroxyvitamin D3 receptor in the immune system. Arch Biochem Biophys 2000; 374: 334–338. 10666315 10.1006/abbi.1999.1605

[76] Nonnecke BJ , Franklin ST , Reinhardt TA , Horst RL . *In vitro* modulation of proliferation and phenotype of resting and mitogen‐stimulated bovine mononuclear leukocytes by 1,25‐dihydroxyvitamin D3. Vet Immunol Immunopathol 1993; 38: 75–89. 7903011 10.1016/0165-2427(93)90114-j

[77] Žofková I , Kancheva RL . The effect of 1,25(OH)_2_ vitamin D3 on CD4^+^/CD8^+^ subsets of T lymphocytes in postmenopausal women. Life Sci 1997; 61: 147–152. 9217273 10.1016/s0024-3205(97)00369-x

[78] Çakmak FN , Erol M , Ergül P , Yalçýner A . T lymphocytes and vitamins. J Pediatr 1999; 135: 531. 10.1016/s0022-3476(99)70185-x 10518094

[79] Phan TX , Jaruga B , Pingle SC , Bandyopadhyay BC , Ahern GP . Intrinsic photosensitivity enhances motility of T lymphocytes. Sci Rep 2016; 6: 39479. 27995987 10.1038/srep39479 PMC5171715

[80] Koenders MA , Giltay EJ , Spijker AT , Hoencamp E , Spinhoven P , Elzinga BM . Stressful life events in bipolar I and II disorder: cause or consequence of mood symptoms? J Affect Disord 2014; 161: 55–64. 24751308 10.1016/j.jad.2014.02.036

[81] Freeman ML , Sheridan BS , Bonneau RH , Hendricks RL . Psychological stress compromises CD8^+^ T cell control of latent herpes simplex virus type 1 infections. J Immunol 2007; 179: 322–328. 17579052 10.4049/jimmunol.179.1.322 PMC2367250

[82] Glaser R , Rice J , Sheridan J *et al* Stress‐related immune suppression: health implications. Brain Behav Immun 1987; 1: 7–20. 2837297 10.1016/0889-1591(87)90002-x

[83] Glaser R , Pearl DK , Kiecolt‐Glaser JK , Malarkey WB . Plasma cortisol levels and reactivation of latent Epstein–Barr virus in response to examination stress. Psychoneuroendocrinology 1994; 19: 765–772. 7991763 10.1016/0306-4530(94)90023-x

[84] Stowe RP , Pierson DL , Feeback DL , Barrett ADT . Stress‐induced reactivation of Epstein–Barr virus in astronauts. Neuroimmunomodulation 2000; 8: 51–58. 10965229 10.1159/000026453

[85] Pakpoor J , Disanto G , Gerber JE *et al* The risk of developing multiple sclerosis in individuals seronegative for Epstein–Barr virus: a meta‐analysis. Mult Scler 2013; 19: 162–166. 22740437 10.1177/1352458512449682

[86] James JA , Kaufman KM , Farris AD , Taylor‐Albert E , Lehman TJA , Harley JB . An increased prevalence of Epstein–Barr virus infection in young patients suggests a possible etiology for systemic lupus erythematosus. J Clin Invest 1997; 100: 3019–3026. 9399948 10.1172/JCI119856 PMC508514

[87] Sokal EM , Hoppenbrouwers K , Vandermeulen C *et al* Recombinant gp350 vaccine for infectious mononucleosis: a phase 2, randomized, double‐blind, placebo‐controlled trial to evaluate the safety, immunogenicity, and efficacy of an Epstein–Barr virus vaccine in healthy young adults. J Infect Dis 2007; 196: 1749–1753. 18190254 10.1086/523813

[88] Haque T , Johannessen I , Dombagoda D *et al* A mouse monoclonal antibody against Epstein–Barr virus envelope glycoprotein 350 prevents infection both *in vitro* and *in vivo* . J Infect Dis 2006; 194: 584–587. 16897655 10.1086/505912

[89] Bu W , Joyce MG , Nguyen H *et al* Immunization with components of the viral fusion apparatus elicits antibodies that neutralize Epstein–Barr virus in B cells and epithelial cells. Immunity 2019; 50: 1305–1316. 30979688 10.1016/j.immuni.2019.03.010 PMC6660903

[90] Messick TE , Smith GR , Soldan SS *et al* Structure‐based design of small‐molecule inhibitors of EBNA1 DNA binding blocks Epstein–Barr virus latent infection and tumor growth. Sci Transl Med 2019; 11: eaau5612. 30842315 10.1126/scitranslmed.aau5612 PMC6936217

[91] Roughan JE , Torgbor C , Thorley‐Lawson DA . Germinal center B cells latently infected with Epstein–Barr virus proliferate extensively but do not increase in number. J Virol 2010; 84: 1158–1168. 19889783 10.1128/JVI.01780-09 PMC2798379

[92] Tabi Z , McCombe PA , Pender MP . Apoptotic elimination of Vβ8.2^+^ cells from the central nervous system during recovery from experimental autoimmune encephalomyelitis induced by the passive transfer of Vβ8.2^+^ encephalitogenic T cells. Eur J Immunol 1994; 24: 2609–2617. 7957554 10.1002/eji.1830241107

[93] Pender MP . Genetically determined failure of activation‐induced apoptosis of autoreactive T cells as a cause of multiple sclerosis. Lancet 1998; 351: 978–981. 9734959 10.1016/S0140-6736(05)60642-3

